# Comparison of Adverse Events between Isolated Left Atrial Appendage Closure and Combined Catheter Ablation

**DOI:** 10.3390/jcm12051824

**Published:** 2023-02-24

**Authors:** Yan Zhang, Jing Yang, Qian Liu, Jinglan Wu, Lei Yin, Jing Lv, Ling You, Yanan Zhang, Lianxia Wang, Yanlei Zhao, Qian Hou, Weilin Jing, Ruiqin Xie

**Affiliations:** 1 First Department of Cardiology, Hebei Institute of Cardiovascular Research, The Second Hospital of Hebei Medical University, Shijiazhuang 050000, China; 2 Second Department of Cardiac Ultrasound, Hebei Institute of Cardiovascular Research, The Second Hospital of Hebei Medical University, Shijiazhuang 050000, China

**Keywords:** atrial fibrillation, percutaneous left atrial appendage closure, catheter ablation, post-procedure adverse events, multivariate analysis

## Abstract

(1) Background: This study aimed to investigate the effect of an additional catheter ablation (CA) procedure on the risk of post-procedure adverse events during CA combined with left atrial appendage closure (LAAC). (2) Methods: From July 2017 to February 2022, data from 361 patients with atrial fibrillation who underwent LAAC at our center were analyzed retrospectively. The adverse events were compared between CA + LAAC and LAAC-only groups. (3) Results: The incidence of device-related thrombus (DRT) and embolic events was significantly lower in the CA + LAAC group than in the LAAC-only group (*p* = 0.01 and 0.04, respectively). A logistic regression analysis revealed that the combined procedure served as a protective factor for DRT (OR = 0.09; 95% confidence interval: 0.01–0.89; *p* = 0.04). Based on a Cox regression analysis, the risk of embolism marginally increased in patients aged ≥65 years (HR = 7.49, 95% CI: 0.85–66.22 *p* = 0.07), whereas the combined procedure was found to be a protective factor (HR = 0.25, 95% CI: 0.07–0.87 *p* = 0.03). Further subgroup and interaction analyses revealed similar results. (4) Conclusions: The combined procedure may be associated with a lower rate of post-procedure DRT and embolization without a higher occurrence of other adverse events after LAAC. A risk-score-based prediction model was conducted, showing a good prediction performance.

## 1. Introduction

The most common cardiac arrhythmia is nonvalvular atrial fibrillation (NVAF). Organ embolism, especially stroke, which is its main complication, has a serious impact on the safety and quality of life of patients with atrial fibrillation (AF). For patients with symptomatic NVAF, catheter ablation (CA) can effectively control and restore sinus rhythm, improving quality of life [1,2]. Although ablation successfully restores sinus rhythm, CA has not been shown to be an effective method of stroke prevention; therefore, patients with AF who are at high risk of stroke should continue long-term oral anticoagulant therapy [3]. Long-term oral anticoagulants, on the contrary, not only increase the risk of bleeding in patients [4] but are also difficult to manage in some elderly patients [5]. Percutaneous transcatheter closure of the left atrial appendage (LAAC) has recently emerged as a safe and effective alternative to long-term anticoagulant therapy [6].

LAAC can be performed as a standalone procedure or in conjunction with CA. CA combined with LAAC (a “one-stop” hybrid procedure) in a single combined procedure can relieve the symptoms of AF via the CA procedure while also effectively preventing the occurrence of thromboembolic events via the LAAC procedure. Thus, the additional risk of multiple procedures can be avoided. In recent years, many observational studies have confirmed the feasibility and effectiveness of the combined procedure [7,8]. However, there have been few studies comparing the combined procedure and isolated LAAC, and the results have been mixed. According to some studies, when compared with a single LAAC procedure, the combined procedure may increase the risk of peridevice leakage (PDL) [9]. However, some studies indicate that the combined procedure does not increase the risk of PDL or other adverse events when compared with LAAC alone [10,11], so the best treatment strategy for LAAC remains unknown. This study aimed to compare the occurrence and influencing factors of adverse events after the combined procedure versus isolated LAAC. The research intended to determine if the additional CA process in the combined procedure increases the risk of adverse events after LAAC and to provide a foundation for further research on the best left atrial appendage occlusion strategy.

## 2. Materials and Methods

### 2.1. Study Design and Study Group

For this retrospective cohort study, we collected and analyzed hospitalization and post-procedure follow-up data from patients with AF who were treated with LAAC in our center from July 2017 to February 2022. The patients who underwent CA in conjunction with LAAC were assigned to the study group (the CA + LACC group), whereas those who underwent LAAC alone were assigned to the control group (the LAAC-only group). The indications for isolated LAAC: age > 18 years; nonvalvular AF; and at least one of the following indications for LAAC: (1) a high CHA2DS2-VASc score (female ≥3, male ≥2) with an increased risk of bleeding (HAS-BLED score ≥ 3), (2) a history of cerebral vascular accident (including stroke and transient ischemic attack) or thromboembolism and under OAC treatment, (3) intolerance to chronic OAC, and (4) a preference for LAAC as an alternative to long-term OAC despite adequate information [12,13]. The contraindication of isolated LAAC: (1) the occurrence of LA thrombus; (2) patients with severe mitral valve disease or sizeable pericardial effusion; and (3) patients who were intolerant to short-term (at least 3 months) OAC due to major bleeding or other contraindications. The indications for CA combined with the LAAC procedure: (1) age > 18 years; (2) symptomatic nonvalvular AF refractory to antiarrhythmic drugs (AADs); (3) accordance with LAAC indications above; and (4) a preference for the procedure of CA combined with LAAC despite adequate information. The contraindication were the same as those for the LAAC-only group and an enlarged LA (>50mm). All participants were included if they met the following criteria: (1) they were over the age of 18 years; (2) they had their first successful “one-stop” procedure or isolated LAAC; and (3) they had complete hospitalization and follow-up information. The exclusion criteria were as follows: (1) LAA occluders were not successfully implanted for a variety of reasons; (2) previous LAAC; and (3) follow-up information was seriously lacking. The research scheme was approved by the Ethics Committee of the Second Hospital of Hebei Medical University (Approval Code: 2020-R128), and all patients provided written informed consent.

### 2.2. CA Procedure

The CA procedure was performed under local anesthesia. Mapping and ablation were performed under the guidance of CARTO (Biosense Webster, Diamond Bar, CA, USA) three-dimensional electroanatomic mapping systems, as well as standard fluoroscopy. The procedure was performed exactly as described previously [14].

### 2.3. LAAC Procedure

The LAAC procedure was performed under local anesthesia and as previously described [15,16]. We ensured that the plug-like occluder (PLO) Watchman (Boston Scientific, Marlborough, MA, USA) devices met the “PASS” (Position, Anchor, Size, Seal) criteria before releasing them. The disk-like occluder (DLO) included the LAmbre (Lifetech Scientific Corp., Shenzhen, China) and Amplatzer Cardiac Plug (ACP) (St. Jude Medical, Saint Paul, MN, USA) devices, which met the “COST” (left Circumflex, Open, Sealing, Tug test) criteria and the “CLOSE” (left Circumflex, Lobe, Orientation, Separation, Elliptical) principle. The patients who underwent the “one-stop” combined procedure repeated the preceding LAAC procedure immediately after CA.

### 2.4. Post-Procedural Anticoagulation

Unless there were contraindications, all patients received OAC therapy for at least 3 months after the procedure. The patients in this study were anticoagulated with Rivaroxaban 20 mg/day (15 mg/day for patients over 75 years of age or with a low body weight) or warfarin, and the international normalized ratio was controlled between 2 and 3 [17]. Dual antiplatelet (Aspirin 100 mg/day and Clopidogrel 75 mg/day) therapy was recommended for another 3 months if the follow-up transesophageal echocardiography (TEE) at 3 months post-procedure showed either no device-related thrombus (DRT) or a limited residual peridevice flow (jet < 5 mm in width) and then Aspirin alone until the 12-month point [18].

### 2.5. Follow-Up

Clinical visits at 3, 6, and 12 months were generally performed. After that, the patients were followed up by a clinical visit or a telephone call every 1 year. Twelve-lead ECGs were obtained at each visit, and serial 24 h Holter monitoring was performed at 3, 6, and 12 months. Additional ECGs or 24 h Holter monitoring was performed in patients who reported symptoms. TEE was performed to assess device occlusion, new peridevice flow, and device-related thrombosis (DRT) at 3 months. AF recurrence was defined as any documented AF lasting >30 s after the blanking period without anti-arrhythmic drugs (AADs) [12]. The loss of follow-up was defined as patients who lost contact after the procedure. The most serious adverse events occurred between the time of the post-procedure and the end of the follow-up period. All-cause death, post-procedure hemorrhage, and major organ embolism/transient ischemic attack (TIA) were the major adverse events in this study, and TEE revealed DRT, PDL, and iatrogenic atrial septal defect (IASD). In this study, the primary endpoint was a composite of the safety and efficacy characteristics of both strategies: (1) ischemic stroke or TIA and (2) significant peri-procedural or device-related complications. The secondary endpoints included an assessment of procedural success and safety, an assessment of bleeding events, the recurrence of AF, and all-cause mortality during the follow-up. TIA is defined as transient neurological symptoms, likely to be due to focal cerebral or ocular ischemia, which last less than 24 h [19]. In our study, the patients with neurological symptoms (e.g., headaches, salivation, limb weakness) were required to have a cerebral MRI or scan, resulting in a diagnosis of stroke or TIA by the neurologist in our center.

### 2.6. Statistical Analyses

Statistical analyses were performed with SPSS version 23.0 (IBM Software Inc, Armonk, New York, NY, USA). Continuous variables are denoted as mean ± standard deviation, whereas categorical variables are denoted as frequency (percentage). For the continuous variables, the t-test was used for intergroup comparisons, and for counting data, the chi-square test or Fisher’s exact test was used. For a multivariable analysis, a Cox or logistic regression model was used. To select the univariate variables for inclusion in the multivariable analysis, a *p* value of 0.1 was used as the cutoff. Moreover, the Kaplan–Meier (KM) curve was analyzed using R software (version 4.2.1). The risk score was calculated using the survival package, and the patients were classified into low-risk and high-risk groups based on the median risk score, with those greater than or equal to the median being classified as high risk. *p* < 0.05 was considered to be statistically significant.

## 3. Results

### 3.1. Study Group

**Figure 1:**
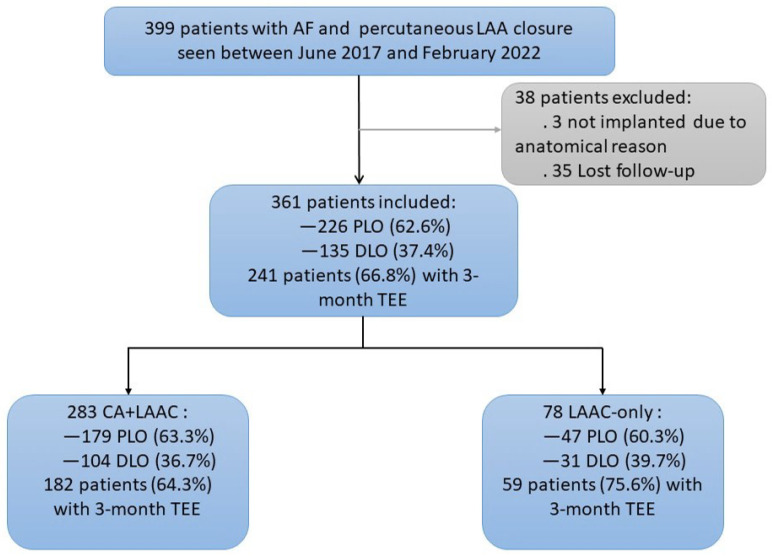
Flowchart of the Study Group.

This study included a total of 399 patients with AF who underwent LAAC at our facility. LAAC was successfully performed in 361 patients (except for in 3 patients in whom LAAC was abandoned owing to anatomical reasons and 35 patients who were not followed up post-procedure). There were 283 patients in the CA + LAAC group and 78 in the LAAC-only group. The average duration of follow-up was 28.27 ± 15.00 months. The follow-up indexes included the incidence of DRT, PDL, and IASD at 3 months after the procedure, as well as the incidence and duration of post-procedure embolism, hemorrhage, and all-cause death. Furthermore, 226 patients had a PLO device (Watchman) inserted, and 135 had a DLO device (124 with LAmbre and 11 with ACP) inserted (Figure 1).

The study cohort between 22 June 2017 and 17 February 2022 is shown. The proportions of the different devices for left atrial appendage (LAA) closure and the patients with a 3-month TEE follow-up are shown, as well as the patients in different groups. AF = atrial fibrillation; CA = catheter ablation; LAAC = left atrial appendage closure; PLO = plug-like occluder; DLO = disc-like occluder; TEE = transesophageal echography.

### 3.2. Baseline Characteristics

Table 1 details the characteristics of the two groups of patients. The diameter of the left atrium was larger in the LAAC-only group than that in the CA + LAAC group (42.12 ± 6.46 mm vs. 39.80 ± 5.05 mm *p* < 0.05), but there was no significant difference in the other baseline characteristics between the two groups. The EHRA symptom scale scores are shown in Table 1 and Supplement Table S1.

### 3.3. Procedural Characteristics and Procedure-Related Complications

There was no significant difference between the two groups in terms of LAAC time and exposure time (Table 2). During the procedure, both groups had no patients with PDLs >3 mm.

There were 14 procedure-related complications in the CA + LAAC group, comprising 5 cases (1.8%) of pericardial effusion resolved with pericardial puncture and 3 cases (1.1%) that resolved spontaneously; 2 cases (0.7%) of groin hematomas absorbed with compression and immobilization; and 4 cases of transient bradycardia after ablation, which improved with temporary pacing therapy. In the LAAC-only group, two patients had a small amount of pericardial effusion that could be absorbed spontaneously (Table 2). There was no statistically significant difference between the two groups in terms of procedure-related complications.

### 3.4. TEE at 3-Month Follow-Up

TEE was performed in a total of 241 patients in the two groups 3 months after the procedure, with 182 cases (64.3%) in the CA + LAAC group and 59 cases (75.6%) in the LAAC-only group. There were no statistically significant differences between the two groups. One patient in the CA + LAAC group had DRT formation, whereas three patients in the LAAC-only group had DRT events, and one patient had left atrial autoradiography. The incidence of DRT was significantly lower in the CA + LAAC group than in the LAAC-only group (0.5% vs. 5.1%, *p* = 0.01) (Table 3). The multivariate logistic regression analysis revealed that the combined procedure was associated with a low risk of DRT formation (OR = 0.09, 95% CI: 0.01–0.89, *p* = 0.04) (Supplementary Figure S1).

PDL >5 mm was not observed in any of the patients with TEE at the 3-month follow-up. There were six cases (3.3%) in the CA + LAAC group and one case (1.7%) in the LAAC-only group with PDL between 3 and 5 mm, with no statistically significant difference between the two groups. Moreover, there was no statistically significant difference in the incidence of IASD between the two groups (Table 3).

### 3.5. Clinical Outcome

In total, 14 adverse events were observed in the CA + LAAC group, comprising 5 cases of embolic events (4 cases (1.4%) of cerebral ischemic stroke and 1 case (0.4%) of asymptomatic pulmonary embolism), 6 cases (2.1%) of bleeding events (one patient experienced cerebral hemorrhage, one skin ecchymosis, and two gingival bleeding with Aspirin, and one experienced epistaxis and one sputum bleeding with Rivaroxaban), and 3 cases (1.1%) of all-cause death. During the follow-up of the LAAC-only group, nine cases of adverse events were documented, comprising four cases of embolic events (5.1%) of ischemic stroke/TIA, one (1.3%) acute myocardial infarction caused by thromboembolism, three cases (3.8%) of bleeding events (one cerebral hemorrhage with Rivaroxaban and two skin ecchymosis with Aspirin), and one case (1.3%) of death due to cerebral hemorrhage (Table 2).

The incidence of embolic events was significantly lower in the CA + LAAC group than that in the LAAC-only group (*p* = 0.04). The incidence of bleeding events and all-cause death did not differ significantly between the two groups (Table 2). Furthermore, the Cox regression analysis of the embolic events in the two groups revealed that the risk of embolism was marginally higher in the patients aged ≥65 years during the follow-up (HR = 7.49, 95% CI: 0.85–66.22 *p* = 0.07), whereas LAAC combined with CA was associated with a lower risk of long-term embolism (HR = 0.25, 95% CI: 0.07–0.87 *p* = 0.03) (Supplementary Table S2). At the same time, subgroup and interaction analyses based on the different procedural groups revealed that the interaction test between procedure and age, sex, and other factors was not statistically significant in the occurrence of post-procedural embolism. This finding also suggests that LAAC combined with CA has a protective effect against the development of embolism (Supplementary Table S3).

**Figure 2:**
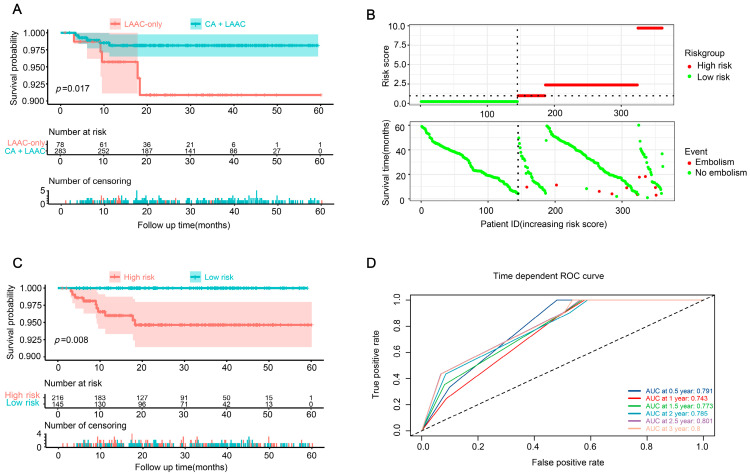
Embolization in patients with AF after procedure. (**A**) The KM survival analysis of patients with post-procedural embolization in groups LAAC-only and CA + LAAC. (**B**) Establishment of a risk scoring model for CSCC; risk score distribution of high-risk group and low-risk group (up), and survival profile of high-risk group and low-risk group (down). (**C**) The KM analysis of high-risk and low-risk populations from (**A**). (**D**) ROC curve analysis of 0.5-year, 1-year, 1.5-year, 2-year, 2.5-year and 3-year overall survival by risk score. CA = catheter ablation; LAAC = left atrial appendage closure; KM = Kaplan–Meier; CSCC = colorectal squamous cell carcinoma; ROC = receiver operating characteristic.

The KM survival analysis of the patients with post-procedural embolization revealed that the patients in the LAAC-only group were more likely to experience embolic events earlier and that they had a poor prognosis (Figure 2A). According to the Cox survival analysis, the procedural strategies and age had an effect on post-procedural embolism. Hence, based on the procedural strategies and age, we used the survival package to calculate the risk score of patients, and then, based on the median risk score, the patients were stratified into low-risk and high-risk groups, with those greater than or equal to the median being classified as high risk. The figure of related risk factors, Figure 2B, shows that patients at a high risk of post-procedural embolism are at high risk, and the KM curve also shows that patients at a high risk have a poor prognosis (Figure 2C). The receiver operator characteristic curve also shows that the risk-score-based prediction model has a good prediction performance for 0.5, 1.0, 1.5, 2.0, 2.5, and 3.0 years after the procedure (Figure 2D).

## 4. Discussion

In patients with AF, LAAC is an alternative to long-term anticoagulation. At least one out of six patients with nonvalvular AF in hospital is strongly recommended to LAAC [20]. When compared with LAAC alone, LAAC combined with CA is not only associated with a lower occurrence of embolic events but also restores sinus rhythm and prevents heart failure caused by AF. The LAA is a complex and highly variable structure, so a comprehensive and personalized evaluation and post-procedure management are needed to reduce the complications of the procedure [21]. However, there is no clarity on whether the combined procedure is harmful when compared with the occlusion of the left atrial appendage. The following are the findings of this study: (1) The risk of procedure-related complications was not higher with the combined procedure. (2) The TEE follow-up results revealed that the risk of DRT was significantly lower in the CA + LAAC group than that in the LAAC-only group and that the incidence rate of PDL was comparable. The combined procedure served as a barrier against thrombus formation on the devices. (3) According to the clinical follow-up results, compared with the LAAC-only group, the risk of embolism was significantly lower in the CA + LAAC group, and there was no significant difference in the risk of bleeding and all-cause death. The combined procedure was a protective factor for embolic events.

1. When compared with the LAAC-only group, the combination of LAAC and CA was not associated with a higher risk of procedure-related complications. In our center, the incidence of acute pericardial effusion requiring interventional treatment was 1.8% in the CA + LAAC group, which is similar to the 1.5% reported in a large multicenter registration study on radiofrequency ablation combined with LAAC [22]. The reason for this could be related to the operator’s extensive experience and skills in performing the procedure.
2. DRT is highly correlated with the occurrence of thromboembolic events, and it is very important to evaluate the occurrence of DRT and analyze the related influencing fac-tors [23]. At the 3-month follow-up, we discovered that the risk of DRT in the patients treated with a one-stop procedure was significantly lower than that in the patients with isolated LAAC. The combined procedure was linked to a lower risk of DRT. This finding can be explained as follows: (1) When compared with LAAC alone, combined CA can restore the sinus rhythm in patients with AF, can improve the blood flow direction in the left atrium, and may reduce the risk of blood cell aggregation. (2) CA can improve left atrial function and accelerate atrial blood flow velocity, and previous studies at our center [15] have confirmed that both the combined procedure and isolated CA can improve left atrial function significantly.

However, the incidence of DRT was 5.1% in the LAAC-only group, which is higher than that observed in other studies [24] (3.4% of DRT after LAAC only). This finding could be attributed to the following factors: (1) In our study, the current anticoagulant therapy may be insufficient for patients with isolated LAAC. According to the most recent AF management guidelines, oral anticoagulants should be combined with single antiplatelet therapy drugs after the procedure in patients with a low bleeding risk from LAAC alone; in patients with a high risk of LAAC, double antiplatelet drugs can be used directly for 1–6 months or until the occluder is completely blocked [12]. Therefore, anticoagulant therapy for patients with LAAC alone can be adjusted based on the risk of bleeding. (2) Two cases of DRT occurred in the LAAC-only group under the condition of regular post-procedural anticoagulation, and the other patient with DRT stopped using anticoagulants within 3 months of the procedure. Taking voluntary withdrawal out of the equation, the incidence of DRT formation in the patients with isolated LAAC was 3.4% (2/59).

We did not observe PDL >3 mm during the LAAC procedure in the CA + LAAC group, but PDL >3 mm was observed in TEE at the 3-month follow-up after the procedure, which may be related to the acute intraprocedural edema of the left atrial ridge (LAR) caused by CA. Previous studies [8,10] have identified that pulmonary venous ridge edema following CA in a one-stop procedure may increase the risk of PDL. However, at the 3-month follow-up, we found that the incidence of PDL was comparable in the two groups. The reason for this could be that edematous LAR in patients undergoing the “one-stop” combined procedure increases the risk of PDL after the procedure. However, because all patients undergoing LAAC in our center were fully rehydrated before it, the average left atrial pressure was maintained at >15 mmHg. Moreover, the LAA was completely filled; hence, its measurement was more accurate, and the selection of the occluder was more suitable. Ryan discovered that the intraoperative saline load increased the size and depth of the LAA orifice during LAAC [25].

3. Although two multicenter studies [22,26] found that combination therapy was effective in reducing stroke, they did not compare it with LAAC only. Bin-Feng Mo [10] noted no significant difference in embolism risk between combined and isolated LAAC, but the sample size was small. During the clinical adverse event follow-up of the two groups of patients in this study, the combined procedure did not increase the risk of bleeding or all-cause death after LAAC and may be associated with the low risk of embolic events. The reason for this could be that the combined procedure not only reduces the risk of thrombus shedding in LAAC but also restores the sinus rhythm in patients with AF. Thus, the normal blood flow direction is restored, and the risk of thrombosis in the atrium and device surface is reduced. Therefore, the occurrence of embolus shedding and embolism events is decreased.

We also found that age ≥65 years is an independent factor influencing the occurrence of embolic events following LAAC. With increasing age, the hemostatic system changes, causing the body to become hypercoagulable and increasing the risk of thrombosis. According to some studies [27], the incidence of venous thrombosis in patients over the age of 65 is three times higher than that in patients aged 45–54 years.

The incidence of adverse events after the procedure in the CA+LAAC group was not higher. A previous study reported that the combined procedure does not increase major adverse cardiovascular events and readmission rates [28], fully demonstrating the safety of the combined procedure. In addition, some studies have shown that left atrial appendage electrical isolation can improve the recurrence of atrial fibrillation after CA in patients with non-paroxysmal atrial fibrillation [29]. Compared with the CA-only procedure, the combined procedure may have an impact on the recurrence of AF after CA, and we need to carry out research to verify this view.

### Limitations

This is a retrospective, single-center study that only reflects a phenomenon that needs to be confirmed via multicentric, prospective large-scale randomized controlled trials in the future.

There were selection biases in our study, including the LAD and use of anticoagulation. Although we had carried out a multivariate analysis and a subgroup analysis, selection biases cannot be completely removed.

The identification of DRT in TEE may have been underestimated because only 66.8% of patients had TEE.

In addition, the embolism events may be underestimated in the population due to the fact that there may be some patients with embolism who were neglected because of being asymptomatic and lacking CT/MRI.

## 5. Conclusions

Additional radiofrequency ablation during the combined procedure is not associated with a higher risk of adverse events following left atrial appendage occlusion, but it may be associated with a reduction in DRT and embolic events, which is a protective factor. Furthermore, the receiver operator characteristic curve also shows that the risk-score-based prediction model that we constructed has a good prediction performance.

## Figures and Tables

**Table 1:** Baseline Characteristics of the Study Population.

	All Patients (*n* = 361)	CA + LAAC (*n* = 283)	LAAC-Only (*n* = 78)	*p* Value (CA + LAAC vs. LAAC-Only)
Age (year)	63.63 ± 8.07	63.71 ± 7.75	63.59 ± 9.21	0.91
Male *n* (%)	213 (59)	166 (58.7)	47 (60.3)	0.80
Medical history				
AF burden				
Paroxysmal AF *n* (%)	117 (32.4)	98 (34.6)	19 (24.4)	0.09
Persistent/Longstanding persistent AF *n* (%)	237 (65.7)	185 (65.4)	59 (75.6)	--
EHRA symptom scale score				
1 point *n* (%)	43 (11.9)	9 (3.2)	35 (44.9)	--
2 point *n* (%)	193 (53.5)	150 (53.0)	43 (55.1)	--
3 point *n* (%)	111 (30.7)	111 (39.2)	--	--
4 point *n* (%)	13 (3.6)	13 (4.6)	--	--
Smoking *n* (%)	60 (16.6)	42 (14.8)	18 (23.1)	0.08
Drinking *n* (%)	55 (15.2)	39 (13.8)	16 (20.5)	0.14
Hypertension *n* (%)	229 (63.4)	185 (65.4)	44 (56.4)	0.15
Diabetes *n* (%)	72 (19.9)	57 (20.1)	15 (19.2)	0.86
Heart failure *n* (%)	251 (69.5)	192 (67.8)	59 (75.6)	0.19
Vascular disease *n* (%)	120 (33.2)	96 (33.9)	24 (30.8)	0.60
CHD *n* (%)	108 (29.9)	85 (30.0)	23 (29.5)	0.93
Ischemic stroke *n* (%)	178 (49.3)	136 (48.1)	42 (53.8)	0.37
Under anticoagulation	111 (62.4)	81 (59.6)	30 (71.4)	0.17
Before anticoagulation	67 (37.6)	55 (40.4)	12 (28.6)	--
Previous bleeding *n* (%)	11 (3.0)	10 (3.5)	1 (1.3)	0.47
LVEF (%)	60.33 ± 8.24	60.57 ± 7.83	59.46 ± 9.60	0.35
LAD (mm)	40.29 ± 5.46	39.80 ± 5.05	42.12 ± 6.46	0.004
LAA velocity (cm/s)	42.15 ± 23.93	42.78 ± 23.85	39.87 ± 24.21	0.34
LAASEC *n* (%)	70 (19.4)	50 (17.7)	20 (25.6)	0.12
CHA2DS2-VASc score	3.88 ± 1.48	3.87 ± 1.47	3.94 ± 1.53	0.73
HAS-BLED score	2.27 ± 1.24	2.28 ± 1.25	2.26 ± 1.21	0.90
BMI (kg/m^2^)	26.50 ± 3.56	26.64 ± 3.65	25.99 ± 3.16	0.16
LAA closure device				0.63
PLO (watchman) *n* (%)	226 (62.6)	179 (63.3)	47 (60.3)	-
DLO *n* (%)	135 (37.4)	104 (36.7)	31 (39.7)	-
LAmbre	124 (91.9)	94 (90.4)	30 (96.8)	-
ACP	11 (8.1)	10 (9.6)	1 (3.2)	-
Antithrombotic therapy at discharge				0.08
Rivaroxaban Tablets	329 (96.1)	275 (97.2)	72 (92.3)	
20mg/day *n* (%)	322 (89.2)	259 (91.5)	63 (80.8)	0.05
15mg/day *n* (%)	25 (6.9)	16 (5.7)	9 (11.5)	-
Warfarin	8 (2.2)	4 (1.4)	4 (5.1)	0.12
Rivaroxaban plus APT	6 (1.7)	4 (1.4)	2 (2.6)	0.61
TEE 3-month follow-up *n* (%)	241 (66.8)	182 (64.3)	59 (75.6)	0.06

Values are mean ± SD or *n* (%). CA = catheter ablation; LAAC = left atrial appendage closure; LAA = left atrial appendage; AF = atrial fibrillation; CHD = coronary heart disease; LVEF = left ventricular ejection fraction; LAD = left atrial diameter; LAASEC = left atrial appendage spontaneous echocardiographic contrast; CHA2DS2-VASc = congestive heart failure, hypertension, age ≥75 years, diabetes mellitus, prior stoke or transient ischemic attack or thromboembolism, vascular disease, age 65 to 74 years, and female sex; HAS-BLED = hypertension, abnormal renal and liver function, stroke, bleeding, labile international normalized ratio, elderly (age < 65 years), drug or alcohol use; BMI = body mass index; PLO = plug-like occlude; DLO = disc-like occlude; ACP = Amplatzer Cardiac Plug; APT = antiplatelet therapy; TEE = transesophageal echography; EHRA = European Heart Rhythm Association. Persistent AF: AF that is continuously sustained beyond 7 days, including episodes terminated by cardioversion (drugs or electrical cardioversion) after ≥7 days. Longstanding persistent AF: continuous AF of >12 months’ duration when decided to adopt a rhythm control strategy.

**Table 2:** Procedural characteristics, procedure-related complications, and follow-up of clinical adverse events.

	CA + LAAC (*n* = 283)	LAAC-Only (*n* = 78)	*p* Value (CA + LAAC vs. LAAC-Only)
Procedural characteristics			
LAAC procedure time, min	49.08 ± 17.40	48.51 ± 20.27	0.85
CA procedure time, min	91.55 ± 28.67	/	-
LAAC fluoroscopy time, s	270.26 ± 190.33	222.26 ± 174.39	0.10
CA fluoroscopy time, s	233.58 ± 199.75	/	-
Complication *n* (%)			
Pericardial effusions			0.32
Pericardial effusions requiring intervention/cardiac tamponade	5 (1.8)	0	0.59
Pericardial effusions not requiring intervention	3 (1.1)	2 (2.6)	0.30
Groin hematoma	2 (0.7)	0	1.00
Temporary pacing	4 (1.4)	/	-
Follow-up events *n* (%)			
Embolism event			0.042
Stroke/TIA	4 (1.4)	4 (5.1)	0.048
AMI	0	1 (1.3)	0.22
Asymptomatic PE	1 (0.4)	0	1.00
Bleeding (brain, gingiva, nose, skin petechiae)	6 (2.1)	3 (3.8)	0.65
Death	3 (1.1)	1 (1.3)	1.00
OAC at the end of follow-up *n* (%)	4 (1.4)	3 (3.8)	0.36
AF recurrence	80 (28.3)	--	--

Values are mean ± SD or *n* (%). CA = catheter ablation; LAAC = left atrial appendage closure; TIA = transient ischemic attacks; AMI = acute myocardial infarction; PE = pulmonary embolism; OAC = oral anticoagulation; AF = atrial fibrillation.

**Table 3:** TEE at 3-month follow-up.

	Overall (*n* = 361)	IN Patients with LAA Imaging (*n* = 241)	CA + LAAC (*n* = 182)	LAAC-Only (*n* = 59)	*p* Value (CA + LAAC vs. LAAC-Only)
DRT *n*(%)	4(1.1)	4(1.7)	1(0.5)	3(5.1)	0.01
PDL *n*(%)					0.83
0 mm	166(45.98)	166(68.9)	126(69.2)	40(67.8)	0.84
0 < PDL ≤ 3 mm	68(18.84)	68(28.2)	50(27.5)	18(30.5)	0.65
3 < PDL < 5 mm	7(1.94)	7(2.9)	6(3.3)	1(1.7)	0.85
≥5 mm	0	0	0	0	-
IASD *n* (%)	53(14.7)	53(22.0)	45(24.7)	8(13.6)	0.07

Values are *n* (%). CA = catheter ablation; LAAC = left atrial appendage closure; LAA = left atrial appendage; DRT = device-related thrombosis; PDL = peridevice leakage; IASD = iatrogenic atrial septal defect.

## Data Availability

The data that support the findings of this study are available in the manuscript and Supplementary Material of this article.

## Supplementary Materials

The following supporting information can be downloaded at: https://www.mdpi.com/article/10.3390/jcm12051824/s1. Table S1. EHRA symptom scale. Table S2. Prognostic factors for overall survival of patients with postoperative embolism event. Table S3. Subgroup analysis for interaction between group and potential covariates in postoperative embolism event. Figure S1. Multivariate logistic regression analysis of DRT.
