# Supplementary material for: Comparison of Adverse Events between Isolated Left Atrial Appendage Closure and Combined Catheter Ablation
Source: J Clin Med. 2023 Feb 24;12(5):1824. doi: 10.3390/jcm12051824 (PMC10003199; doi:10.3390/jcm12051824)
Supplement: Supplementary file 1 [file jcm-12-01824-s001.zip › jcm-2127240-supplementary.pdf]

Supplementary information

Comparison of Adverse Events Between Isolated Left Atrial Appendage Closure and Combined Catheter Ablation

Yan Zhang [1], Jing Yang [1], Qian Liu [1], Jinglan Wu [2], Lei Yin [1], Jing Lv [1], Ling You [1], Yanan Zhang [1], Lianxia Wang [1], Yanlei Zhao [1], Qian Hou [1], Weilin Jing [1] and Ruiqin Xie [1,*]

[1] First Department of Cardiology, Hebei Institute of Cardiovascular Research, The Second Hospital of Hebei Medical University, Shijiazhuang 050000, China; zhangyan@hebmu.edu.cn (Y.Z.); yangjing85@hebmu.edu.cn (J.Y.); 105335@hebmu.edu.cn (Q.L.); yinlei20213396@stu.hebmu.edu.cn (L.Y.); qihang2021@vip.163.com (J.L.); youling@hebmu.edu.cn (L.Y.); zhangyanan@hebmu.edu.cn (Y.Z.); wanglianxia@hebmu.edu.cn (L.W.); zyl@hebmu.edu.cn (Y.Z.); houqian@hebmu.edu.cn (Q.H.); 20193352@stu.hebmu.edu.cn (W.J.)

[2] Second Department of Cardiac Ultrasound, Hebei Institute of Cardiovascular Research, The Second Hospital of Hebei Medical University, Shijiazhuang 050000, China; 906098672.doc.wu@hebmu.edu.cn

* Correspondence: xieruiqin66@163.com or xieruiqin@hebmu.edu.cn

FUNDING

This research was funded by S&T Program of Hebei (No.20377730D) and the Natural Science Foundation of Hebei Province (H2020206646).

CONFLICTS OF INTEREST

The authors declare no conflicts of interest.

DATA AVAILABILITY STATEMENT

The data that supports the findings of this study are available in the manuscript and supplementary material of this article.

Supplementary Table S1
EHRA symptom scale

Score	Symptoms	Description
**1**	None	AF does not cause any symptoms
**2a**	Mild	Normal daily activity not affected by symptoms related to AF
**2b**	Moderate	Normal daily activity not affected by symptoms related to AF, but patient

		troubled by symptoms
**3**	Severe	Normal daily activity affected by symptoms related to AF
**4**	Disabling	Normal daily activity discontinued
**Six symptoms, including palpitations, fatigue, dizziness, dyspnoea, chest pain, and anxiety during AF, are evaluated with regard to how it affects the patient's daily activity, ranging from none to symptom frequency or severity that leads to a discontinuation of daily activities. AF = atrial fibrillation; EHRA = European Heart Rhythm Association.**		

Supplementary Table S2
Prognostic factors for overall survival of patients with postoperative embolism event

	Univariate analysis			Multivariate analysis		
**Characteristics**	HR	(95%CI)	***P* Value**	HR	(95%CI)	***P* Value**
**Group**	0.25	0.07 - 0.85	0.015	0.252	0.073 - 0.874	0.0298
**Gender**	3.34	0.86 - 12.93	0.081	-	-	-
**Age**	9.65	1.22 - 76.17	0.032	7.488	0.847 - 66.219	0.0702
**Type of AF**	1.46	0.41 - 5.16	0.561	-	-	-
**LVEF**	0	0 - Inf	0.998	-	-	-
**LAD**	84815246.98	0 - Inf	0.998	-	-	-
**LAA velocity**	0.47	0.13 - 1.67	0.246	-	-	-
**LAASEC**	1.63	0.42 - 6.31	0.478	-	-	-
**LAAC device**	0.41	0.09 - 1.93	0.26	-	-	-
**BMI**	0.71	0.2 - 2.53	0.6	-	-	-
**CHA2DS2-VASc score**	1.51	1.02 - 2.22	0.038	1.18	0.770-1.808	0.4467
**HAS-BLED score**	1.59	0.93 - 2.74	0.09	-	-	-
**Smoking**	0	0 - Inf	0.998	-	-	-
**Drinking**	0.62	0.08 - 4.91	0.653	-	-	-
**Previous bleeding**	0	0 - Inf	0.998	-	-	-
**Hypertension**	0.87	0.24 - 3.07	0.824	-	-	-
**Diabetes**	0.47	0.06 - 3.7	0.473	-	-	-
**HF**	0.94	0.24 - 3.63	0.925	-	-	-
**CHD**	1.13	0.29 - 4.37	0.862	-	-	-
**Stroke/TIA**	2.69	0.69 - 10.4	0.153	-	-	-

Group：CA+LAAC vs. LAAC-only；Gender：female vs male；Age(y): ≥65 vs. ＜65；Type of AF：paroxysmal AF vs. persistent/longstanding persistent AF；LVEF (%): <50 vs. ≥50; LAD (mm):≥35 vs. ＜35；LAA velocity（cm/s）：≤40 vs. ＞40; LAA closure device: DLO vs. PLO; BMI（kg/m^2）:≥25 vs. ＜25; LAASEC、Smoking、Drinking、Previous bleeding、Hypertension、diabetes、HF、CHD、Ischemic stroke: Yes vs. No. AF= atrial fibrillation; LVEF= left ventricular ejection fraction；LAD= Left atrial diameter；LAASEC= Left atrial appendage spontaneous echocardiographic contrast；LAA= left atrial appendage; LAAC= left atrial appendage closure；BMI= body mass index；HF= heart failure; CHD= coronary heart disease; TIA= transient ischemic attacks.

Supplementary Table S3
Subgroup analysis for interaction between group and potential covariates in postoperative embolism event

Characteristics	N	HR(95%CI)	*P* value for interaction
**Gender**			0.560
**female**	213	0.124 (0.011-1.386)	-
**male**	148	0.31 (0.069-1.392)	-
**Age**			0.224
**＜65**	186	0.000 (0.000-Inf)	-
**≥65**	175	0.305 (0.082-1.137)	-
**Type of AF**			0.543
**persistent/longstanding persistent AF**	244	0.297 (0.060-1.475)	-
**paroxysmal AF**	117	0.154 (0.022-1.103)	-
**LVEF**			1
**≥50%**	316	0.233 (0.067-0.809)	-
**＜50%**	45	NA	-
**LAD**			1
**＜35mm**	50	NA	-
**≥35mm**	311	0.255 (0.074-0.884)	-
**LAA velocity**			0.658
**＞40cm/s**	153	0.175 (0.035-0.875)	-
**≤40cm/s**	208	0.305 (0.043-2.169)	-
**LAASEC**			0.196
**NO**	287	0.152 (0.034-0.684)	-
**YES**	74	0.926 (0.084-10.229)	-
**BMI**			0.147
**<25kg/m^2**	117	0.863 (0.090-8.298)	-
**≥25kg/m^2**	244	0.11 (0.020-0.606)	-
**Smoking**			1
**NO**	301	0.219 (0.063-0.758)	-

**YES**	60	NA	-
**Drinking**			0.297
**NO**	306	0.286 (0.076-1.067)	-
**YES**	55	0.000 (0.000-Inf)	-
**Previous bleeding**			0.999
**NO**	350	0.252 (0.073-0.872)	-
**YES**	11	NA	-
**Hypertension**			0.308
**NO**	132	0.104 (0.011-0.998)	-
**YES**	229	0.441 (0.08-2.418)	-
**Diabetes**			0.237
**NO**	290	0.199 (0.053-0.745)	-
**YES**	71	Inf (0.000-Inf)	-
**HF**			0.711
**NO**	109	0.297 (0.026-3.401)	-
**YES**	252	0.241 (0.048-0.958)	-
**CHD**			0.527
**NO**	253	0.185 (0.041-0.829)	-
**YES**	108	0.543 (0.049-5.994)	-
**Stroke/TIA**			0.574
**NO**	183	0.442 (0.040-4.887)	-
**YES**	178	0.204 (0.045-0.920)	-

AF= atrial fibrillation; LVEF= left ventricular ejection fraction； LAD= Left atrial diameter； LAASEC= Left atrial appendage spontaneous echocardiographic contrast; LAA= left atrial appendage; LAAC= left atrial appendage closure； BMI= body mass index； HF= heart failure; CHD= coronary heart disease; TIA= transient ischemic attacks.

Supplementary Figure S1
Multivariate logistic regression analysis of DRT

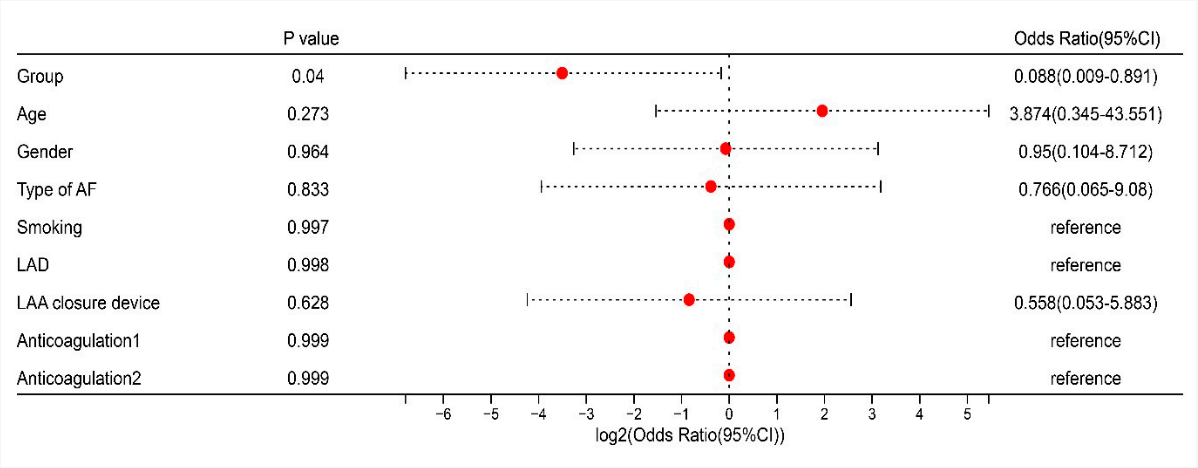

Group：CA+LAAC vs. LAAC-only；Age(y): ≥65 vs ＜65；Gender：female vs. male； Type of AF：paroxysmal AF vs. persistent/longstanding persistent AF；Smoking: Yes vs. No; LAD (mm):≥35 vs.＜35；LAA closure device: DLO vs. PLO; Anticoagulation 1: warfarin vs. Rivaroxaban; Anticoagulation 2: Rivaroxaban plus APT vs. Rivaroxaban; Reference: 0-Inf. DRT= device-related thrombosis; AF= atrial fibrillation; LAD= Left atrial diameter； LAA= Left atrial appendage; APT=antiplatelet therapy, Inf= infinity.
